# Cluster categorization of urban roads to optimize their noise monitoring

**DOI:** 10.1007/s10661-015-4994-4

**Published:** 2015-12-12

**Authors:** G. Zambon, R. Benocci, G. Brambilla

**Affiliations:** Department of Earth and Environmental Sciences, Università degli Studi di Milano, Bicocca, Piazza dalla Scienza 1, 20126 Milan, Italy; Istituto di Acustica e Sensoristica “Orso Mario Corbino”, CNR, Via del Fosso del Cavaliere 100, 00133 Rome, Italy

**Keywords:** Road noise, Urban area, Monitoring techniques, Statistical analysis

## Abstract

Road traffic in urban areas is recognized to be associated with urban mobility and public health, and it is often the main source of noise pollution. Lately, noise maps have been considered a powerful tool to estimate the population exposure to environmental noise, but they need to be validated by measured noise data. The project Dynamic Acoustic Mapping (DYNAMAP), co-funded in the framework of the LIFE 2013 program, is aimed to develop a statistically based method to optimize the choice and the number of monitoring sites and to automate the noise mapping update using the data retrieved from a low-cost monitoring network. Indeed, the first objective should improve the spatial sampling based on the legislative road classification, as this classification is mainly based on the geometrical characteristics of the road, rather than its noise emission. The present paper describes the statistical approach of the methodology under development and the results of its preliminary application to a limited sample of roads in the city of Milan. The resulting categorization of roads, based on clustering the 24-h hourly *L*_Aeqh_, looks promising to optimize the spatial sampling of noise monitoring toward a description of the noise pollution due to complex urban road networks more efficient than that based on the legislative road classification.

## Introduction

Road traffic in urban areas is one of the key issues for a variety of aspects associated with municipal, regional, and national policies due to its strong connection with urban mobility and public health. In particular, the Directive 2002/49/EC of the European Parliament and of the Council has addressed a common approach aimed at avoiding, preventing, and reducing the harmful effects of exposure to environmental noise (Directive 2002, ec.europa 2015). Road traffic is the main source of noise in urban areas, and its assessment and management is, therefore, strictly linked to the aforementioned issues. For this reason, monitoring traffic and noise in urban areas has been the object of many studies (i.e.,Fidel 1978, Brown and Lam 1987, Kumar and Jain 1994, Miedema and Vos 1998, Garcia and Faus 1991, Fields 1993, Barrigon et al. 2005). and their results have been used to construct noise maps to estimate the relevant population exposure.

To draw noise maps, both numerical models of outdoor sound propagation and monitoring stations are used, the latter usually located near receivers where sound levels are dominated by specific sources. Noise maps updating is achieved scaling the noise levels of pre-calculated (basic) noise maps as a function of the difference observed between measured and calculated original grid data.

The process of linking the output of sound level meters to noise calculation models in order to produce automatic and updated noise maps was applied some years ago. For instance, in 2003, Madrid Environmental Administration, together with Brüel & Kjær, decided to develop a new concept of data post-processing, based on dynamic noise maps (SADMAM, Sistema Actualización Dinámica Mapa Acústico and Spain (2006)). In the same years, Paris authorities published dynamic noise maps on internet. In this case, the noise maps were and are still currently fed by road traffic noise measurements on a daily basis (Bruitparif 2015).

At present, software developers implementing acoustic models provide application modules to link sound level meters with their acoustic simulation software. This operation, although extremely appealing from a technical perspective, requires large investment, due to the cost of the devices currently available to measure sound levels.

The need of developing and implementing a low cost monitoring network was tackled by another interesting experience in the project SENSEable (SENSEable PISA 2011). This idea is going to be further developed in the recently approved DYNAMAP (Dynamic Acoustic Mapping), an EU co-funded project in the LIFE 2013 framework (DYNAMAP - LIFE13 ENV/IT/001254). The project is aimed at developing dynamic noise maps through customized low-cost monitoring devices for data collection, their transmission to central processing server to calculate maps scaling through the implementation of a GIS-based software application in order to reduce calculation load. To develop and validate the methodology, two pilot areas have been identified: the first one is in the city of Milan and covers a significant portion of the city, including different types of roads and acoustical scenarios. The second pilot area is located along a busy motorway, namely the ring road enclosing the city of Rome. Sensors will be installed in hot spots where traffic counting is unavailable to feed the dynamic mapping system with real-time information on noise levels.

The present paper deals with the preliminary study carried out in the city of Milan to provide a scheme of the approach to be applied throughout the DYNAMAP project. The approach briefly consists in applying to a sample of roads, a statistical analysis with the aim of grouping together roads with similar 24-h noise time patterns, in associating each cluster time pattern with a non-acoustic characteristics (e.g., vehicle flow rate) and then extend the cluster classification (connection to a specific group) to the rest of un-sampled roads according to the available non-acoustic characteristic of each road. Thus, each un-sampled road can be allocated to a specific group (cluster).

The preliminary data sample refers to 58 sites corresponding to 8 legislative road classes. Their characteristics are described in “Acoustic database” section. As an alternative to the legislative road classes, “Statistical analysis” section describes the reduced number of road types obtained by the cluster analysis, having homogeneous noise level patterns over the day and night periods to be used as a reference. Each cluster is also cross-checked by non-acoustic information (e.g., rush hour traffic flow or average daily traffic volume) in order to assign road segments to the relevant clusters. The statistical fundamentals applied to optimize the number of monitoring sites are described in “Stratified sampling by legislative road classification and by mean cluster profiles” section. This issue related to spatial sampling has a significant impact on future investment costs. “Estimate of daytime *L*_Aeqd_ from the hourly L_Aeqh_” section deals with temporal sampling within the 24 h. This technique offers the advantage, by reducing the measurement time, to save resources which can be fruitfully used to improve the resolution of spatial sampling. The values of the noise descriptors on medium or long term estimated by those measured at shorter sampling time are affected by uncertainty. Studies on this aspect have been carried out in Alberola et al. (2005), Bellucci et al. (2003). , Proceedings Euronoise (2003), Bordone and Sacerdote (1977). Makarewicz (2012). Gaja et al.(2003), Heiß (2013)). Discussion and conclusions are reported in Discussion and “Conclusions” sections, respectively.

## Acoustic database

The dataset considered in the present work refers to road traffic noise in the city of Milan, Italy, and is formed by 138 patterns of 24-h continuous monitoring of the hourly equivalent levels, *L*_Aeqh_, in 58 different sites, belonging to eight legislative road classes, named A (motorways), D (thoroughfare roads), E (district roads), and F (local roads), the last two divided into two and four sub-groups, respectively. In the present study, the sub-groups in E and F classes were merged. In the road network of Milan, the road A length is 3.3 %, that of D roads is 2.0 %, E roads have a length of 13.8 %, and local roads (F) are the largest percentage (61.0 %).

Data were recorded on weekdays and in the absence of rain, as required by the Italian decree D.M. Ambiente 16/3/1998 (Decreto Ministero dell’Ambiente 16 marzo 1998). Because of the non-homogeneity of *L*_Aeqh_ level dataset, due to different monitoring conditions, such as different distances from the road and also to the configuration of the street itself (its geometry, the presence of reflecting surfaces and obstacles along sound propagation and types of paving), each *i*th hourly *L*_Aeqhij_ level of the *j*th 24-h pattern was referred to its corresponding daytime level, *L*_Aeqdj_, as follows:

1 $$ {\updelta}_{ij}={L}_{{\mathrm{Aeqh}}_{ij}}\hbox{-} {L}_{\mathrm{Aeqdj}}\ \left[\mathrm{dB}\right]\ \left(i= 1,\ ..........,\  24\ h;\ j= 1,\ ..........,\  58\right) $$

**Fig. 1:**
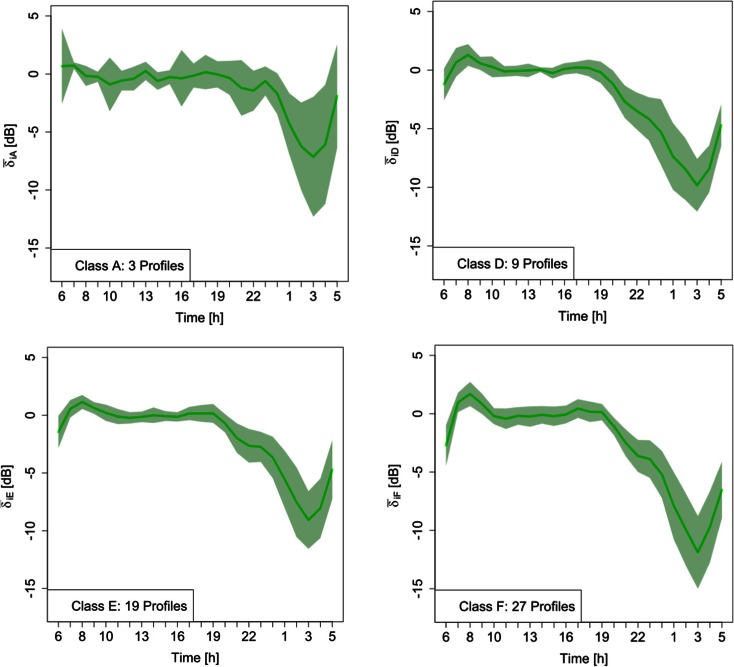
24-h mean patterns ${\overline{\delta}}_{im}$ (*green line*) and the corresponding ± the standard error of the mean for each legislative road class (*light green area*)

The daytime level, *L*_Aeqd_, was chosen as reference for the hourly *L*_Aeqh_ because this descriptor is more often available than the nighttime value, *L*_Aeqn_. For all the 58 sites, the vehicle flow rate at rush hour (time interval 7:30–8:30 a.m.) was available too. In 32 sites, where the monitoring extended over more days, the median of the *δ*_*ij*_ hourly values was considered, as this parameter is less influenced by the presence of outliers. Figure 1 shows the 24-h mean pattern ${\overline{\delta}}_{im}$(green line, with *m* = A, D, E, F) and the corresponding ± the standard error of the mean for each legislative road class (light green area).

## Statistical analysis

In addition to the legislative road classification, it was chosen to classify roads according to statistic criteria applied to their noise emission. Generally, such emission depends on the road activity, its use in the urban context, its width, the presence of reflecting surfaces, obstacles, type of paving, etc.. Some of these parameters are considered in the legislative road classification, but this classification very often does not reflect the actual use of the roads. For a more adequate description of the real temporal behavior of noise in complex urban scenarios, such as the mobility network of the city of Milan, the issue was faced considering an agglomeration method to search for similarities among the 24-h continuous monitoring of the hourly equivalent *L*_Aeqh_ levels. After computing the *δ*_*ij*_ values, the corresponding time pattern provides a tool to group together roads following the same vehicular dynamics, therefore allowing a more adequate description of such road networks rather than that obtained by the legislative road classification. For this reason, unsupervised clustering algorithms were applied to group together patterns found to be “similar” to one another. Various algorithms (hierarchical agglomeration using Ward algorithm (Ward 1963). K-means algorithm (Hartigan and Wong 1979), Partitioning Around Medoids (Kaufman and Rousseeuw 1990). Expectation Maximization algorithm implemented by the “mclust” module (Fraley 2012) were considered, and their results compared. The number of clusters was chosen as a compromise between satisfactory discrimination and the need to limit the number of groups. The range of solutions for clustering was set from four groups (for a straightforward comparison with the legislative road classes considered) to two (corresponding to the minimal discrimination). Euclidean distance was chosen as the metric of the distance among observations. The statistical software R, an open-source software environment for statistical computing and graphics, was applied for the clustering (http://www.r-project.org/). A detailed discussion on the statistical analysis is given in Zambon et al. (2014) where a validating test of the clustering results was carried out by the “clValid” package (Brock et al. 2008) in order to assess the quality of the clustering and assign a score to the different clustering algorithms. The results obtained by the best performing algorithms were further tested to check for their independence, confirming the robustness of the obtained clusters.

The resulting two clusters were formed of roads belonging to different legislative classes, as reported in Table 1. For the four-group solution, which is directly comparable to the legislative road classes, a poor matching is observed between road and cluster partitioning. The F class roads are distributed over all the four groups, whereas the remaining classes are distributed in the first two groups. This confirms that the road traffic is primarily linked to the effective urban mobility rather than its legislative classification, as shown also by the outcomes of previous studies, such as in Brambilla and Gallo (2010). where the monitoring data of road traffic noise collected in 244 sites distributed in 37 Italian cities were analyzed by K-means clustering.

**Table 1:** Composition of the two clusters according to the legislative road classes

Cluster	Legislative road class				Total
	A	D	E	F	
1	1 (33.3 %)	5 (55.6 %)	6 (31.6 %)	19 (70.4 %)	31 (53 %)
2	2 (66.7 %)	4 (44.4 %)	13 (68.4 %)	8 (29.6 %)	27 (47 %)

**Fig. 2:**
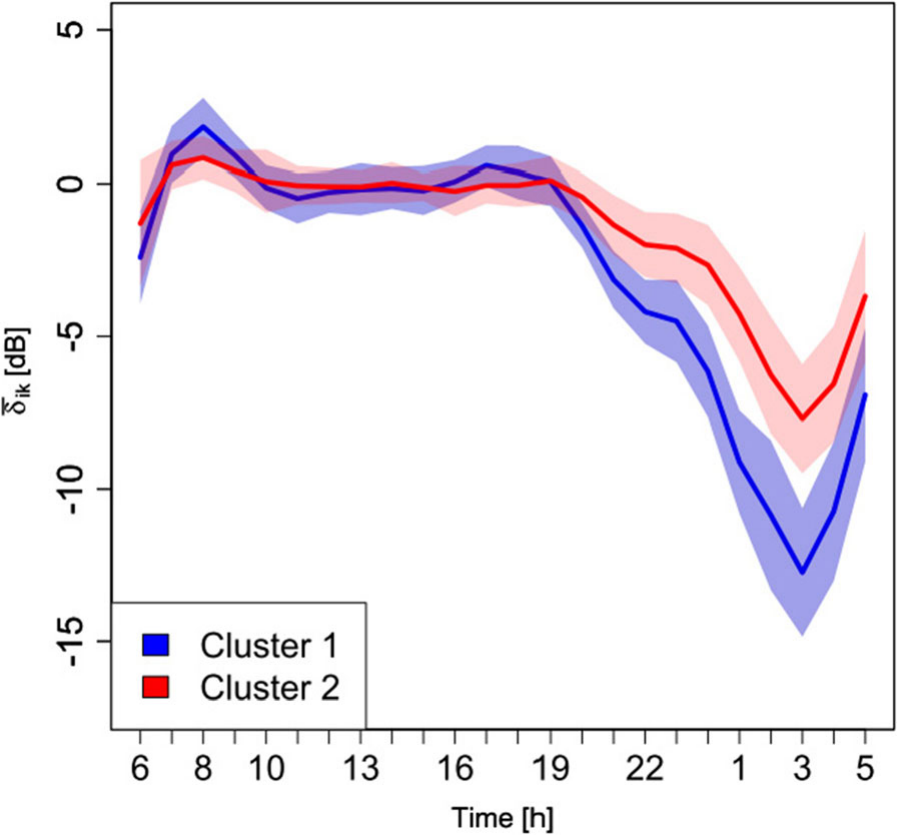
Mean cluster profiles, ${\overline{\delta}}_{ik}$, and the corresponding ± standard deviation

The two-cluster solution represents a satisfying balance between an adequate differentiation among time patterns and the need to get a solution easy to be applied. As shown in Table 1, the two clusters are formed primarily of the contributions from different temporal profiles belonging to roads of F class for cluster 1 (made up of 31 temporal profiles corresponding to 53 % of total) and to roads of class A and E for cluster 2 (made up of 27 temporal profiles corresponding to 47 % of total). Class D roads are almost equally distributed over the two clusters. This result confirms that the noise time patterns are not directly linked to the legislative road classification. Figure 2, which accounts for the two-cluster solution reported in Table 1, shows the profiles of mean cluster values, ${\overline{\delta}}_{ik}$ with *k* = 1, 2, and the corresponding ± the standard error of the mean for each cluster. Cluster 1 (blue line) presents two peaks: the first at the hourly interval 8–9 and the second at 17 h. It remains close to *L*_Aeqd_ until 19 h, afterwards, it goes down in the night period till 3 h and then it starts raising again. Cluster 2 (red line) shows just one lower peak at 8–9 h and higher values at nighttime. In the remaining time period, it shows a similar behavior of cluster 1.

By the above clusters, it is possible to estimate the daytime *L*_Aeqd_ and nighttime *L*_Aeqn_ levels from the value of a single hourly *L*_Aeqh_.

**Fig. 3:**
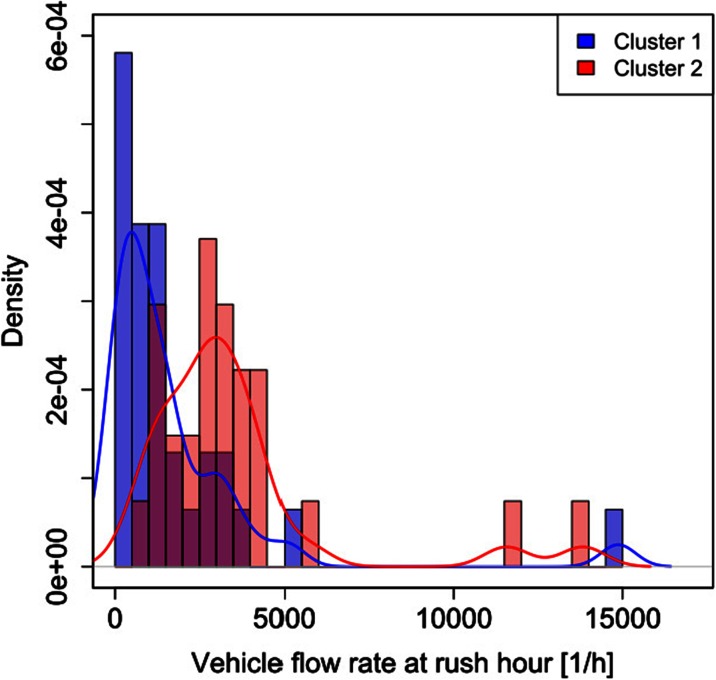
Histogram and probability density vs. vehicle flow rate at rush hour for the K-means two-cluster results

**Fig. 4:**
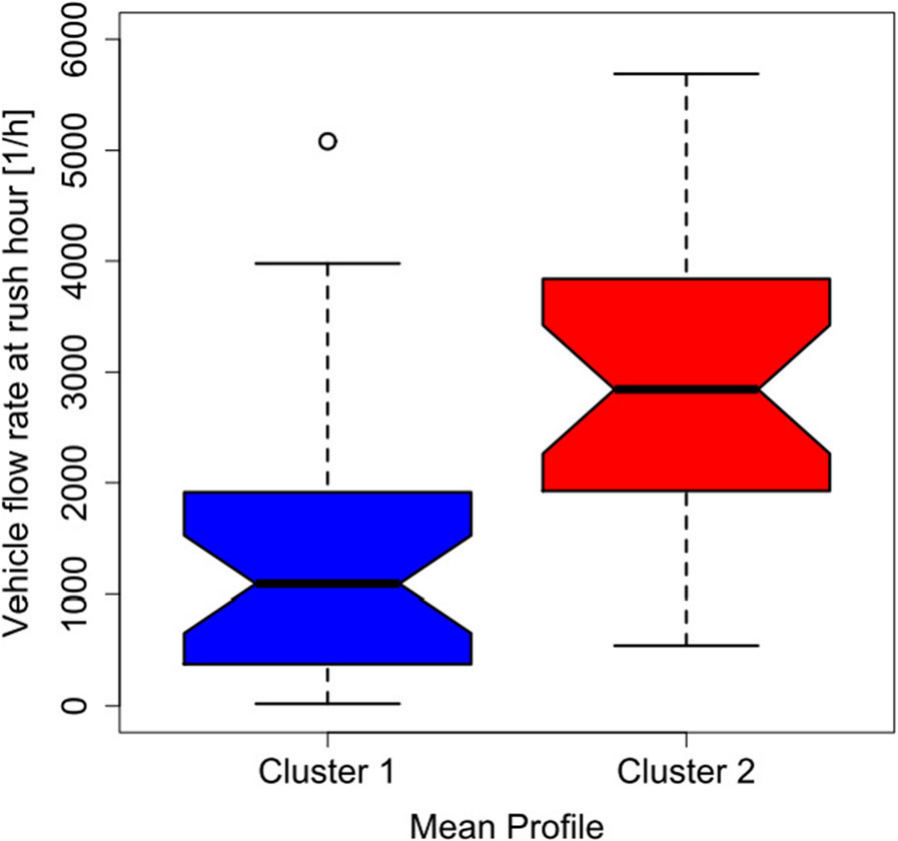
Box plots of the traffic flow rate at rush hour for the two mean cluster profiles

**Fig. 5:**
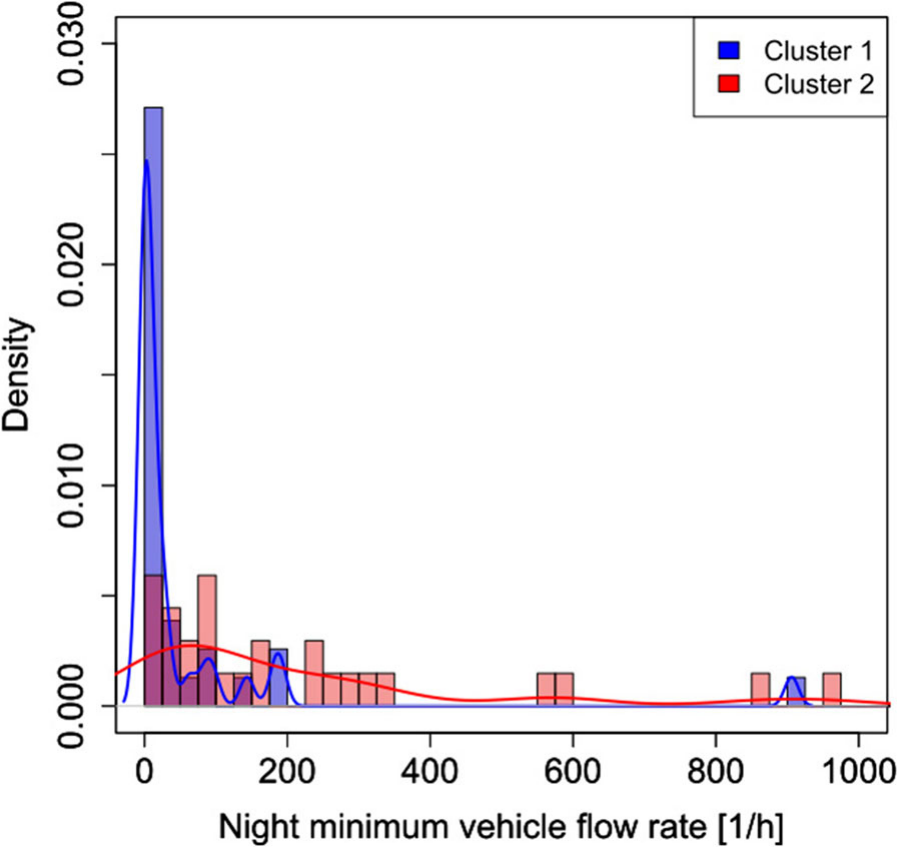
Histogram and probability density vs. night minimum vehicle flow rate for the K-means two-cluster results

**Fig. 6:**
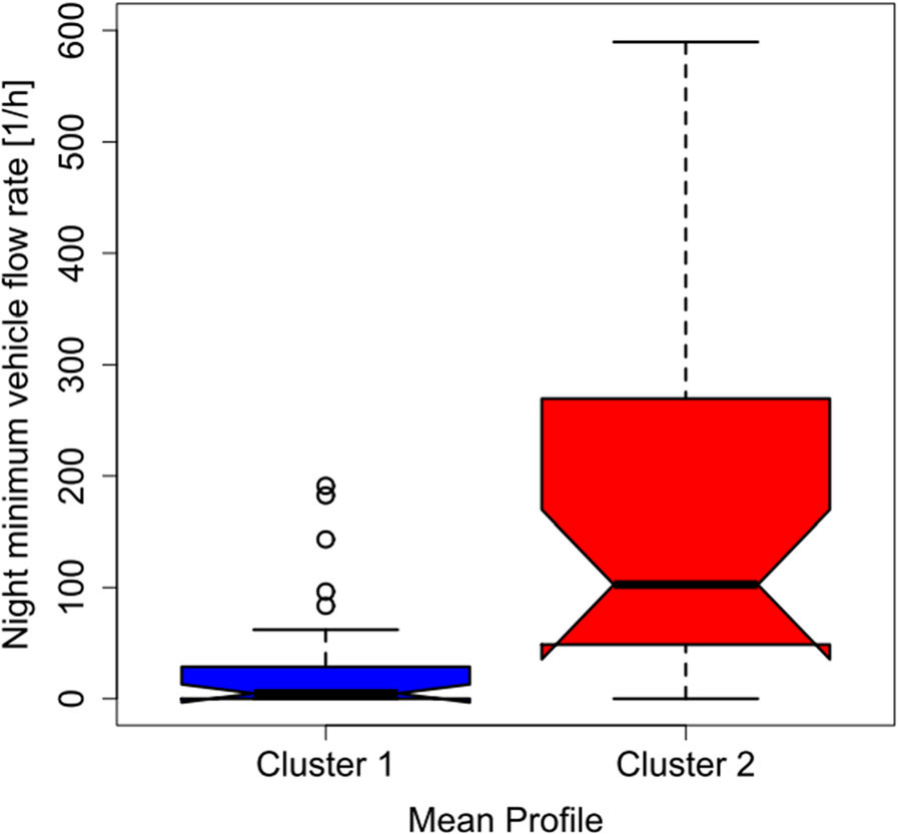
Box plots of the night minimum vehicle flow rate for the two mean cluster profiles

Unlike the legislative classification of roads, the two obtained cluster profiles cannot be applied in a straightforward way without any indication linking them to a specific feature. Such limitation can be overcome by associating each mean cluster profile with the corresponding traffic flow rate at rush hour and with the minimum vehicle flow rate for each of the 58 roads. The traffic data were provided by the AMAT agency, in charge of the traffic mobility management of the city of Milan.^1^ Figures 3, 4, 5, and 6 show the probability density and the box plots of the vehicle flow rate at rush hour for the two mean cluster profiles. In particular, the density distributions are quite separate between the two clusters (Fig. 3), and their interquartile range does not overlap (Fig. 4). Thus, a vehicular flow rate at rush hour of 2000 vehicles per hour can be considered as threshold between the two profiles, that is roads featuring higher values (>2000 vehicles/h) can be associated with cluster 2, whereas lower flow rates (<2000 vehicles/h) can be allocated to cluster 1. For the night minimum vehicle flow rate parameter, the density distributions show different behaviors: cluster 1 shows a sharp profile centered around zero vehicles per hour, whereas cluster 2 shows a flatter distribution though peaked at higher values (Fig. 5). The corresponding box plot shows a clear distinction between the interquartile ranges for the two clusters (Fig. 6). In this case, the threshold value between the clusters is around 40 vehicles per hour.

## Stratified sampling by legislative road classification and by mean cluster profiles

Further the categorization of roads by clustering, a general method to determine and optimize the number of sites to be monitored for the calibration of the acoustic model and for the updating of noise maps is needed. For this purpose, the spatial stratified sampling was selected to reduce the number of measurement points required for the above calibration. By and large, in spatial stratified sampling, the sample can often be split up into sub-samples. This has the advantage to decrease the variance of each sub-sample, to use partly non-random methods applied to sub-groups/clusters or to study strata individually (Kish 1965). Regarding road traffic noise, the stratified sampling is often applied, as reported in Brown and Lam (1987) and has recently been applied in Barrigon et al. (2005) and tested in Barrigón et al. (2011). For instance, in Barrigón et al. (2011). it was found that the categorization method yielded similar results for the overall analysis of the city to those obtained with the value of the points of the grid method with considerably fewer sampling points. The categorization method also seems to be a more suitable predictor for new measurements, particularly for levels in the noisiest streets of the town.

As well known, to estimate the mean of the population within a predefined accuracy ± *E*, the minimum number of elements of a sample, *n*_min_, is calculated by (for *n*_min_ < 30)

2 $$ {n}_{\min }=\frac{t_{n-1,\alpha}^2\cdot {s}^2}{E^2} $$

where *t*_*n* − 1,*α*_^2^ is the value of the Student's *t* distribution for a confidence level (*1*−*α*) and *ν* = (*n*−*1*) number of observations and *s* is the sample standard deviation.

In general, applying Eq. (2) to the four legislative road classes is not straightforward because usually the sample standard deviation *s* of sound levels for each road class is unknown, though its value could be estimated as proposed in Pihur et al. (2007) and in Van Bell (2008)). In the dataset under study, the measured *s* values and the mean daytime *L*_Aeqd_ and nighttime *L*_Aeqn_ levels were considered. Because of the aforementioned non-homogeneity of the dataset levels measured in different environmental conditions, each *j*th value was referred to the 24-h *L*_Aeq24j_ reference value:

3 $$ {\varDelta}_{mj} = {L}_{\mathrm{Aeq}\mathrm{mj}}\hbox{--}\ {L}_{\mathrm{Aeq}24\mathrm{j}}\kern1em \left(m = d,\ n;\ j = 1, \dots \dots ..,\  58\right) $$

where *d* and *n* stand for the day and night period. Considering an accuracy *E* = ± 0.5 dB for the estimate of the referred mean $\overline{\varDelta_d}$ and $\overline{\varDelta_n}$, Eq. (2) provides the minimum sample dimension, *n*_min_, required. The results shown in Table 2 show that the data collected for the legislative road classes D, E, and F are enough to estimate the mean referred daytime level, $\overline{\varDelta_d}$, within the fixed accuracy *E* as they exceed *n*_min_, whereas they are insufficient for roads of class A, as well as to estimate the mean referred nighttime level, $\overline{\varDelta_n}$, for A and D roads. Thus, for the considered level of accuracy *E* = ±0.5 dB, more measurements are required. The minimum sample dimension, *n*_min_, strongly depends on the variability of collected data and, therefore, it would be recommended to choose representative sites with a high variability (high *s* values) of sound levels.

**Table 2:** Comparison between the minimum sample dimension, *n*_min_, as calculated for the functional classification of roads and the statistical profiles according to Eq. (2) assuming an accuracy *E* = ±0.5 dB

Road class	No. of 24-h monitoring periods	Minimum sample dimension *n*_min_	
		$\overline{\varDelta_d}$	$\overline{\varDelta_n}$
A	5	7	214
D	20	4	90
E	54	2	34
F	59	1	50
Cluster 1	71	2	38
Cluster 2	67	1	19

As highlighted above, there was not a complete matching between the mean 24-h profiles obtained by the legislative classification of roads and the ones determined by the cluster analysis. Thus, the minimum sample dimension, *n*_min_, for each mean cluster profile was calculated and reported in Table 2. The results show that the collected data enable the mean referred daytime, $\overline{\varDelta_d}$, and nighttime, $\overline{\varDelta_n}$, levels to be properly estimated within the given accuracy, *E* = ±0.5 dB (*n* > *n*_min_).

The redistribution of temporal profiles, operated by the clustering algorithm, boosts the efficiency because of a better correspondence between the statistical profiles and the measured sound sources than the one observed for the legislative classification of roads.

## Estimate of daytime *L*_Aeqd_ from the hourly *L*_Aeqh_

Another important issue deals with the temporal sampling of sound levels. By means of this technique, the noise descriptors on medium or long term are estimated by their values measured at shorter sampling time (Utley 1982). The reduced time length may represent a good compromise between the need of sample accuracy and costs of monitoring. However, the estimated values are affected by uncertainty, whose amount depends on the ratio between the measurement time and the medium long-term time, as well as on the variability of the noise immission at the measurement point; on this issue for instance, see Makarewicz and Gałuszkab (2012), Can et al. (2011), and Brocolini et al. (2013).

In the present study, the measured value of a single hourly *L*_Aeqh_ has been considered to estimate the daytime (from 6 to 22 h) equivalent level *L*_Aeqd_, the exact value of which is given by

4 $$ {L}_{\mathrm{Aeqd}}=10\times \log \left[\frac{1}{16}{\displaystyle \sum_{i=1}^{16}{10}^{0.1{L}_{\mathrm{Aeqhi}}}}\right]\kern0.48em \left[\mathrm{dB}\left(\mathrm{A}\right)\right] $$

The hourly interval providing the most accurate estimate of *L*_Aeqd_ can be identified among those ones satisfying the following condition for *δ*_*ij*_ and the sample standard deviation, *s*:

5 $$ \left\{\begin{array}{c}\hfill {\delta}_{ij}\hfill \\ {}\hfill s\hfill \end{array}\to 0\ \left(\kern0.24em i\kern-0.29em =\kern-0.24em 1\kern0.24em \mathrm{h}, \dots \dots \dots, \kern0.20em 24\kern0.32em \mathrm{h};\kern0.20em j\kern-0.24em =\kern-0.28em 1,\kern0.32em \dots \dots \dots, \kern0.44em 58\right)\right.. $$

The present analysis is limited to 35 sites out of 58, namely 19 out of 31 for cluster 1 and 13 out of 27 for cluster 2. A further criterion can be based on the probability, *P*, that the error of *L*_Aeqd_ estimate, calculated by the mean profile, lies in a predefined accuracy interval, *ε*. We considered two accuracy intervals, namely *ε* = ±0.50 dB and *ε* = ±0.75 dB. For each road class, *m*, (with *m* = A, D, E, F) and *i*th hourly interval, firstly, we evaluated the difference, $\left({\overline{\delta}}_{im}-{\delta}_{ijm}\right)$, between the mean profile, ${\overline{\delta}}_{im}$, and the corresponding *j*th profile, *δ*_*ijm*_, and then we calculated the ratio, *P*_*m*_, between the differences falling within the selected accuracy interval, *ε*, and the total number of profiles in each road class, *N*_*jm*_:

6 $$ {P}_m=\frac{N_{\left({\overline{\updelta}}_{im}\hbox{-} {\updelta}_{ijm}\right)\le \upvarepsilon}}{N_{jm}}\cdot 100\ \left[\%\right] $$

**Fig. 7:**
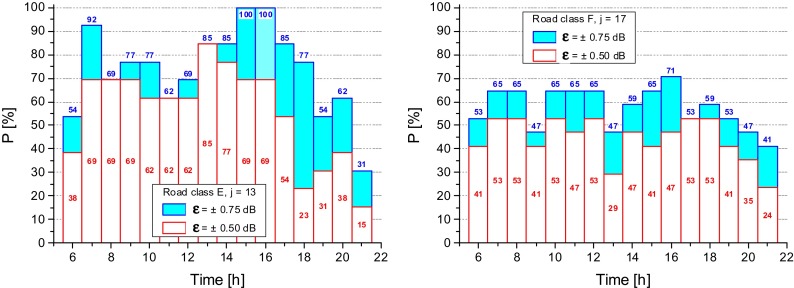
Probability, *P*, that the error of the estimated *L*_Aeqd_ lies within the accuracy intervals, *ε* = ±0.50 dB and *ε* = ±0.75 dB

In Fig. 7, the calculated probability values, *P*_*m*_, that is the probability, in percentage, that the errors of the estimated *L*_Aeqd_ fall within the selected accuracy interval, *ε*, are shown for E and F road classes. The results for A and D classes are not reported due to their poor sample size. The hourly intervals providing the most accurate estimate of *L*_Aeqd_ level can be easily identified, that is those with the highest values of P. For local road (type F) values of *P* lower than those corresponding to the E class are observed. As expected, a wider accuracy interval (*ε* = ±0.75 dB) yields higher probability, *P*_*m*_.

**Fig. 8:**
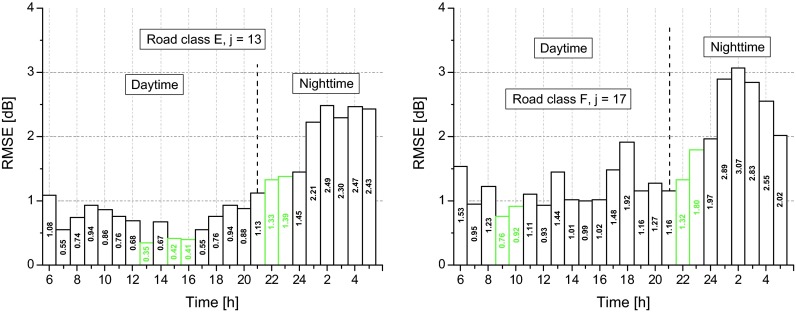
Root mean square error, RMSE_*im*_, of the estimate of *L*_Aeqd_ levels. The hourly intervals with the lowest RMSE_*im*_ values for the daytime and nighttime periods are reported in green

For each road class, *m*, and *i*th time interval, the root mean square error, RMSE_*im*_, was calculated too:

7 $$ {\mathrm{RMSE}}_{im}=\sqrt{\frac{{\displaystyle \sum {\left({\overline{\updelta}}_{im}\hbox{-} {\updelta}_{ijm}\right)}^2}}{N_{jm}}}\ \left[\mathrm{dB}\right] $$

where $\left({\overline{\delta}}_{im}-{\delta}_{ijm}\right)$ is the difference, for the *i*^*th*^ time interval, between the mean profile, ${\overline{\delta}}_{im}$, and the correspondent single profile, *δ*_*ijm*_. Figure 8 shows the calculated RMSE_*im*_ values, which are higher during the night period, with peaks in the hourly intervals between 2 and 4 h. The hourly intervals with the lowest *RMSE*_*im*_ values both for the daytime (6–22 h) and nighttime (22–6 h) periods are reported in green.

**Fig. 9:**
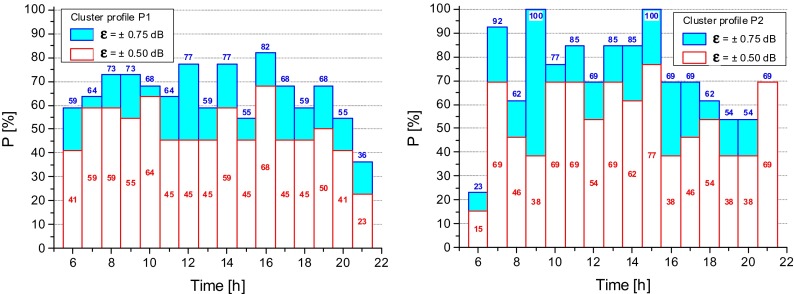
Probability, *P*_*ik*_, that the error of the estimate of *L*_Aeqd_ falls within the accuracy intervals, *ε* = ±0.50 dB and *ε* = ±0.75 dB

The above analysis was applied also to the two mean cluster profiles, *k*, (with *k* = P1, P2). Figure 9 shows for each profile the probability, *P*_*ik*_, that the error on the calculated estimate of *L*_Aeqd_ falls within the assigned accuracy interval, *ε*.

**Fig. 10:**
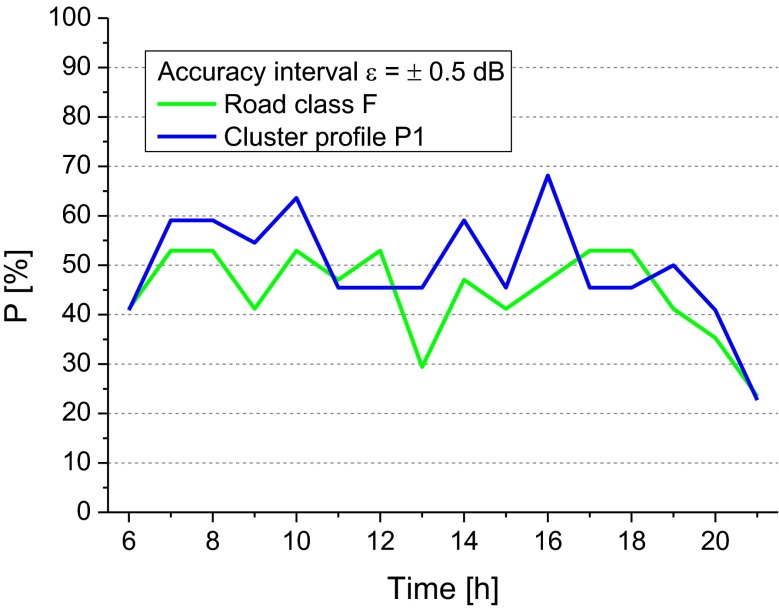
Probability, *P*_*ik*_, that the error of the estimate of *L*_Aeqd_ falls within the accuracy intervals *ε* = ±0.50 dB obtained for the road class F and the cluster profile P1

Because cluster profile P1 is largely formed by roads belonging to class F (70.4 %, see Table 1), the obtained probability values *P*_*i*_ for the cluster P1 and legislative road class F have been compared and shown in Fig. 10 for the accuracy interval *ε* = ±0.50 dB. It can be seen that for the majority of the hourly intervals the cluster profile P1 provides higher *P*_*i*_ values than those given by the road class F.

**Fig. 11:**
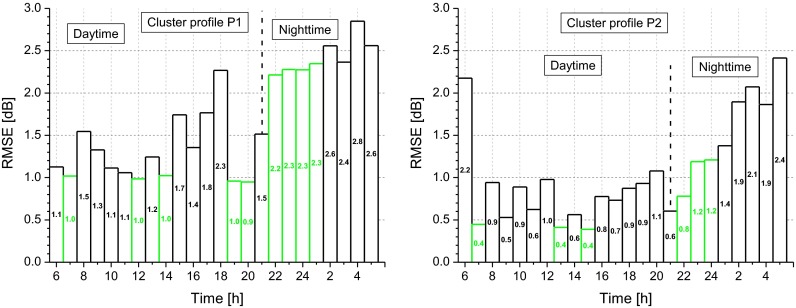
Root mean square error, RMSE_ik_, of the estimate of *L*_Aeqd_ levels. The hourly intervals with the lowest RMSE_*ik*_ values for the daytime and nighttime periods are reported in green

For each profile, *k*, and *i*th time interval, we calculated the RMSE_*ik*_ by Eq. (7). The results, reported in Fig. 11, show generally higher RMSE_*ik*_ values during the night period. The hourly intervals more suitable for estimating the daytime levels, *L*_Aeqd_, are highlighted in green.

## Discussion

The database used to set up the proposed procedure for classifying roads in urban areas based on their noise immission is rather limited in number of sites and refers to the city of Milan only. Thus, it can be considered only as a preliminary step toward collecting a wider data set more representative of the road traffic reality in Milan.

Notwithstanding, the cluster profile procedure performs better than that based on legislative road classification and, at least for the estimate of the mean value of *L*_Aeqn_ − *L*_Aeq24_, the proposed procedure looks to be promising, as it requires a lower number of monitoring sites than those demanded by the legislative road classification.

Of course, due to the nature of current samples, the results at this stage cannot be generalized to the entire road network of Milan and, even more, to other cities. For this reason, road traffic noise monitoring is still in progress to enlarge the database and refining the results to improve their statistical robustness.

Thus, the proposed procedure should be viewed as a methodological approach, hopefully to stimulate its further applications in other cities, also looking at differences and similarities.

## Conclusions

The cluster analysis applied to the 24-h continuous monitoring of the hourly equivalent levels *L*_Aeqh_ using the hierarchical method was confirmed by K-means and PAM algorithms. It showed that the dataset of measurements can be suitably grouped into two mean profiles to be applied to roads with vehicular flow rate greater (cluster 2) and less (cluster 1) than 2000 vehicles per hour at rush hour and the threshold value is around 40 vehicles per hour for the night minimum vehicle flow rate.

Dealing with the minimum sample size for the spatial stratified sampling, the two clusters were more efficient to estimate of the mean *L*_Aeqd_ and *L*_Aeqn_ levels, as they required less monitoring sites than those obtained from the legislative road classification because of a better correspondence between the statistical profiles and the measured sound sources. Such trend profiles represent a tool to group together roads following the same vehicular dynamics, therefore allowing a more real description of complex road networks and a proper description of the noise indicators considered compared to the legislative road classification.

In terms of temporal sampling, the procedure may represent a good compromise between the need of sample accuracy and costs investment of the monitoring campaign. Considering the hourly intervals suitable for an accurate estimate of the daytime equivalent level, *L*_Aeqd_, those between 11 and 15 h and between 22 and 24 h are the most appropriate for each legislative road class with a RMSE_*im*_ between about 0.5 and 1.5 dB and between about 1 and 2 dB, respectively. The same was observed for the two-cluster solution, as for the hourly intervals between 11 and 15 h and between 22 and 24 h, the obtained RMSE_*ik*_ was between about 0.5 and 1.5 dB and between about 1 and 2.5 dB, respectively.

## Footnotes

1 ^*^http://www.amat-mi.it/it/mobilita/dati-strumenti-tecnologie/
